# Comparison of the Shear Bond Strength Using Primers with Different Application Numbers on Dental Zirconia

**DOI:** 10.1055/s-0043-1777821

**Published:** 2024-07-16

**Authors:** Suphakit Opatragoon, Awiruth Klaisiri, Tool Sriamporn, Niyom Thamrongananskul

**Affiliations:** 1 Department of Prosthodontics, Faculty of Dentistry, Chulalongkorn University, Bangkok, Thailand; 2 Division of Restorative Dentistry, Faculty of Dentistry, Thammasat University, Pathum Thani, Thailand; 3 Department of Prosthodontics, College of Dental Medicine, Rangsit University, Pathum Thani, Thailand

**Keywords:** zirconia primer, phosphate-containing primer, primer application, shear bond strength

## Abstract

**Objective** This study examined the effect of the number of phosphate-containing primer applications on the shear bond strength (SBS) of zirconia to resin cement.

**Materials and Methods** 315 square specimens (10 × 10 × 4 mm ^3^ ) were manufactured from Cercon ht presintered zirconia blocks. Alumina particles were used to sandblast zirconia specimens. These specimens were randomly divided into six primer-based groups: No primer application (NP), CLEARFIL CERAMIC PRIMER (C), PANAVIA V5 Tooth Primer (T), M&C PRIMER (MC), Monobond N (MN), and Z-PRIME plus (Z), and then separated into application number (1–4) groups (excluding NP). Each specimen was bonded with resin cement. The SBS was measured using a universal testing machine. The debonded surface was examined with a stereomicroscope.

**Statistical Analysis** The SBSs were analyzed using two-way analysis of variance.

**Results** Applying the primer twice exhibited the highest SBSs in each group, with significant differences in the T, MN, and Z groups. However, the SBS in the MC group was significantly lower on the second application. One-hundred percent adhesive failure was observed in all groups.

**Conclusion** Within the limitations of this study, prior to cementation, the sandblasted zirconia surface should be applied twice with a phosphate-containing primer other than MC to maximize the SBS at the zirconia-resin cement interface.

## Introduction

Zirconia, also known as zirconium dioxide (ZrO _2_ ), has been used as a dental material in recent years. In addition to its esthetic appeal, biocompatibility, and practical application, zirconia possesses various mechanical properties, including high flexural strength and toughness. 1 2 3 Zirconia is used in fabricating posts and cores, dental implants, orthodontic brackets, and fixed-partial dentures. 2 3 However, bonding between other materials, such as resin cements and dental zirconia, is challenging. This is due to the chemical inertness and absence of silica in dental zirconia. 4

Currently, zirconia cement is used as a resin cement or methacrylate-based luting cement. Furthermore, it has been demonstrated that the structural integrity of zirconia reduces its bonding effectiveness with resin cement. Zirconia is acid-resistant and structurally inert because it typically contains a higher crystalline phase concentration than other ceramics. To strengthen the bond between resin cement and zirconia, a variety of mechanical and chemical surface treatment techniques have been performed. 4 Surface preparation techniques, including grinding, tribochemical silica coating, chemical etching, selective infiltration etching, laser irradiation, and chemical vapor deposition, have been used. These procedures, whether used individually or in conjunction with one another, are required to form a long-lasting bond between composite luting cement and zirconia. 5

Various compositions of zirconia can be chemically bonded using couplers as the connecting mechanism. Given the absence of silica in the substrate, the application of a silane-coupling agent in conjunction with aluminum oxide sandblasting results in a low bond strength. When using silane coupling agents, the application of a tribochemical silica coating allows high-strength ceramics based on alumina and zirconia to be chemically more reactive with the resin. 5 6 This essentially increases the resin bond strength values. Additionally, using primers and luting agents containing a phosphate monomer, such as 10-methacryloyloxydecyl dihydrogen phosphonate (10-MDP) 7 8 and a zirconate coupler agent has been recommended in conjunction with airborne particle abrasion (aluminum oxide or silica-coating). The combination of these primers with airborne particle abrasion produces a stronger bond. 9 10 11

Using sandblasting to achieve a mechanical bond and then applying a zirconia primer is the most common method for achieving a strong zirconia bond. 10 Mahgoli et al discovered that a single application of zirconia primer improved the bonding capability of the zirconia-resin composite. 12 Furthermore, Klaisiri et al reported that multiple primer applications during zirconia bonding strengthened the bond. 13 Due to the increased amount of functional monomer after application, additional applications of zirconia primer may have strengthened the zirconia's chemical bonding. 13 14 15 However, there is no report on whether the number of applications of each brand of MDP-containing primer affects the bonding ability of zirconia-resin cement. Therefore, the purpose of this study was to determine how the number of times each MDP-containing primer brand was applied affects the shear bond strength (SBS) of the following: CLEARFIL CERAMIC PRIMER (Kuraray Noritake Dental Inc., Aichi, Japan), M&C PRIMER (Sun Medical Co., Shiga, Japan), Monobond N (Ivoclar Vivadent Inc., Schaan, Liechtenstein), PANAVIA V5 Tooth Primer (Kuraray Noritake Dental Inc., Aichi, Japan), Z-PRIME plus (Bisco Inc., Schaumburg, German). The null hypothesis of the study was that multiple applications of each brand of phosphate-containing primer have no effect on the SBS of zirconia and resin cement.

## Materials and Methods

### Specimen Preparation, Fabrication, and Characterization

**Fig. 1:**
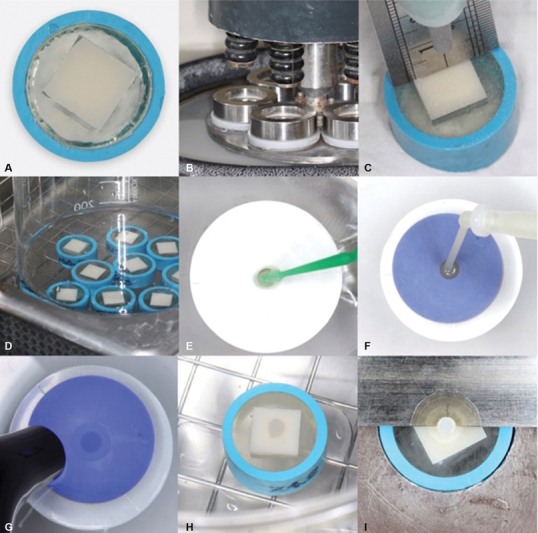
Method: ( **A** ) Embedded in clear auto-polymerizing acrylic resin in a polyvinylchloride tube; ( **B** ) Polished with 400 to 1200 grit SiC paper using a polishing machine at 2 kg/cm ^2^ force, 100 rpm; ( **C** ) Air-abraded for 10 s/cm ^2^ at 0.2 MPa with 110 μm Al _2_ O _3_ particles at a perpendicular (90 degrees) distance of 10 mm controlled by a stainless steel ruler; ( **D** ) Cleaned for 10 minutes in an ultrasonic bath with 99% isopropanol; ( **E** ) Primer application; ( **F** ) Injected with PANAVIA V5 Paste in a 2.8 **-** mm diameter silicone mold; ( **G** ) light-cured for 40 seconds with an LED light-curing unit with an intensity of 1,100 mW/cm ^2^ and a 405 nm wavelength; ( **H** ) Stored in distilled water for 24 hours at 37°C; ( **I** ) Evaluated using a universal testing machine with a 0.5 mm notch-edge crosshead blade.

In this investigation, 315 square specimens (10 × 10 × 4 mm ^3^ ) were manufactured from Cercon ht presintered zirconia blocks (Dentsply Sirona, Bensheim, Germany). The zirconia specimens were polished with 1,200 grit silicon carbide paper using a polishing machine (MINITECH 233, PRESI, France) and then sintered at the manufacturer-recommended temperatures before the furnace was gradually cooled to room temperature. Each specimen was placed in a polyvinylchloride tube, embedded in a clear autopolymerizing acrylic resin ( Fig. 1A ), and polished with 400 to 1,200 grit silicon carbide paper using a polishing machine (MINITECH 233, PRESI, France) at 2 kg/cm ^2^ force, 100 rpm, and submerged in running water for 120 seconds ( Fig. 1B ). 13

A sandblasting unit (Basic Quattro, Renfert, Germany) was used to air-abrade each specimen for 10 s/cm ^2^ using 110 microns Al _2_ O _3_ particles at a pressure of 0.2 MPa and a perpendicular (90 degrees) distance of 10 mm between the air-abrasion instrument and the target surface controlled by a stainless steel ruler ( Fig. 1C ). 16 The specimens were cleaned for 10 minutes in an ultrasonic bath (VGT-1990QTD, China) with 99% isopropanol ( Fig. 1D ).

### Primer Application

According to ISO 4049:2009 and ISO 29022:2013, the specimens were cemented in controlled bonding areas. To obtain the maximum resin cement bonding area, an adhesive tape (thickness = 50 microns) with a 2.38-mm diameter inner circular hole was prepared with a laser die cutting machine and the adhesive tape was applied to each specimen and cutting the tape's side produced a circular hole that was easy to remove. The specimens were randomly divided into six groups based on the primer, and each group was subdivided into four application number groups (except the control group) (n = 15; “ *n* ” for control group and each subgroups) as follows:

**Group 1** ; Control group; no primer applied (NP)

**Group 2** ; CLEARFIL CERAMIC PRIMER applied 1–4 times (C1-C4; Kuraray Noritake, Japan)

**Group 3** ; M&C PRIMER applied 1–4 times (MC1-MC4; Sun Medical Co., Shiga, Japan)

**Group 4** ; Monobond N applied 1–4 times (MN1-MN4; Ivoclar Vivadent Inc., Schaan, Liechtenstein)

**Group 5** ; PANAVIA V5 Tooth primer applied 1–4 times (T1-T4; Kuraray Noritake Dental Inc., Aichi, Japan)

**Group 6** ; Z-PRIME plus applied 1–4 times (Z1-Z4; Bisco, Inc., Schaumburg, German).

Ten microliters of each primer were placed on disposable microbrushes (Applicator tips, Dentsply DeTrey, Konstanz, Germany). The primers were applied to the bonding area of each specimen for 2 seconds ( Fig. 1E ). After 1 minute, oil/water–free air was blown on the specimen from a mobile dental unit using a triple syringe (Mobile dental unit, Thai Dental Products, Bangkok, Thailand) with a pressure of 40 to 50 psi at a distance of approximately 10 mm from the zirconia surface for 10 seconds. Until each group had received the required number of primer applications, the procedure was repeated.

### Cementation Procedure

The specimens were injected with PANAVIA V5 Paste (Dual-cured, Self-etching, Shade Universal (A2), Kuraray Noritake, Japan) in a 2.8 **-** mm diameter silicone mold ( Fig. 1F ) and light-cured for 40 seconds perpendicular to the template and as close as possible using an LED light-curing unit (Demi Plus, Kerr, United States) with an intensity of 1,100 mW/cm ^2^ and a 405 nm wavelength ( Fig. 1G ). The specimens were light cured for another 40 seconds perpendicular to the resin cement on each side. The adhesive tape was removed from the specimens and stored in distilled water for 24 hours at 37°C ( Fig. 1H ). Table 1 lists the materials and resin cements used in this study.

**Table 1:** Materials, abbreviations, manufacturers, compositions, and batch numbers

Material	Composition	Lot number	Manufacturer
Cercon ht	3 mol% Y-TZP: Yttrium oxide 5%, Hafnium oxide < 3%, aluminum oxide, silicon oxide < 1%	18032881	Dentsply Sirona Inc., Bensheim, Germany
PANAVIA V5 Tooth Primer	2-hydroxyethyl methacrylate 25-50%, 10-methacryloyloxydecyl dihydrogen phosphate, hydrophilic aliphatic dimethacrylate, accelerators, water	8S0097	Kuraray Noritake Dental Inc., Aichi, Japan
CLEARFIL CERAMIC PRIMER	Ethanol >80%, 3-trimethoxysilylpropyl methacrylate <5%, 10-methacryloyloxydecyl dihydrogen phosphate	8T0067	Kuraray Noritake Dental Inc., Aichi, Japan
M&C PRIMER	M&C PRIMER A: Methyl methacrylate 60–70%, acetone 20–30%, MDP, VTD M&C PRIMER B: Methyl methacrylate 10-97%, 1-Propanol, 3-(trimethoxysilyl)-, methacrylate 0-20%	FE3123	Sun Medical Co., Shiga, Japan
Monobond N	Ethanol 50–100%, water, silane methacrylate, phosphoric acid methacrylate 1– < 2.5%, sulfide methacrylate	W85744	Ivoclar Vivadent Inc., Schaan, Liechtenstein
Z-PRIME plus	Ethanol 75–85%, Bis-GMA 5–10%, 2-hydroxyethyl methacrylate 5–10%, 10-MDP 1–5%	2100007374	Bisco Inc., Schaumburg, German
PANAVIA V5 Paste, Shade Universal (A2)	Paste: Bisphenol A diglycidylmethacrylate 5–15%, TEGDMA <5%, silanated barium glass filler, silanated fluoroaluminosilicate glass filler, colloidal silica, surface treated aluminum oxide filler, hydrophobic aromatic dimethacrylate, hydrophilic aliphatic dimethacrylate, dl-camphorquinone, initiators, accelerators, pigments	3Q0053	Kuraray Noritake Dental Inc., Aichi, Japan

### Bond Strength Testing

The specimens were evaluated using a universal testing machine (EZ-S, SHIMADZU, Japan) with a 0.5 mm notch-edge crosshead blade ( Fig. 1I ) at a speed of 1 mm/min in accordance with ISO 29022:2013. The equation R = F/A, where F was the load for specimen failure (N) and A was the cross-sectional interfacial area, was used to compute the shear bond strength R (MPa) (mm ^2^ ).

### Failure Evaluation

A stereomicroscope (SZ 61, OLYMPUS, Japan) was used to examine the zirconia surfaces after the specimens were debonded to categorize the failure modes as adhesive, cohesive, or mixed failure. Cohesive failure was defined as failure within the resin cement, adhesive failure was defined as failure at the interface of the specimens and resin cement, and mixed failure was defined as a combination of adhesive and cohesive failure.

### Statistical Analysis

The quantitative data from the 6 independent groups were analyzed using BM SPSS Statistics 25.0 (SPSS Inc., Chicago, Illinois, United States) at a 95% confidence level. The Kolmogorov–Smirnov test at a significance level of 0.05 was used to determine normality. Levene's test was then used to analyze the equality of variation. The results showed that the data had normal distribution and equal variance. Therefore, two-way analysis of variance was used to analyze the data, followed by least significant difference test ( *p* < 0.05).

### Determination of Phosphorous Concentration by ICP-OES

We investigated the correlation between MDP concentration and bond strength. The amount of phosphorus in each brand of primer that contains phosphate was defined as the amount of MDP. The concentration of phosphorous was measured by inductively coupled plasma-optical emission spectrometry (ICP-OES model 7300DV, PerkinElmer, UK). One μL of each phosphate-containing primer brand was dissolved in 30 mL deionized water. Each solution was directly placed into the ICE-OES from the centrifuge tube of each group. The instrument was calibrated using a phosphorous standard solution (PerkinElmer, UK) that was diluted to prepare a set of standards. The standard curves were obtained by comparing the concentration of each standard in milligrams per liter (mg/L) to its intensity (counts). Each unknown sample's concentration was determined using the standard curves. Three separate measurements were performed.

## Results

In this investigation, no specimens prematurely debonded prior to testing the shear bond strength.

The shear bond strength means and ranges are presented in Table 2 . The results indicated that the T group had the strongest shear bond strength at all application numbers, and was significantly different compared with the other groups ( *p* < 0.05). The Control group had the lowest shear bond strength at all application numbers, which was significantly different compared with the other groups ( *p* < 0.05).

**Table 2:** Mean shear bond strength, standard deviation (MPa), and percentage of failure mode

Groups	Applications	Mean shear bond strength (SD)	Percentage of failure mode adhesive
NP group (control)	−	1.145(0.20)	100
C group (CLEARFIL CERAMIC PRIMER)	1	13.437(1.19) ^(a)^	100
	2	14.096(1.19) ^(a,A)^	100
	3	10.124(0.99) ^(b,B)^	100
	4	8.930(1.09) ^(b)^	100
MC group (M&C PRIMER)	1	10.258(1.65) ^(C)^	100
	2	8.618(1.45)	100
	3	6.336(1.28) ^(c)^	100
	4	5.447(0.99) ^(c)^	100
MN group (Monobond N)	1	11.521(1.29) ^(C)^	100
	2	16.791(1.52)	100
	3	15.177(1.33) ^(d)^	100
	4	14.375(1.31) ^(d)^	100
T group PANAVIA V5 Tooth Primer	1	19.679(1.54) ^(e)^	100
	2	25.161(1.67)	100
	3	21.804(1.87) ^(f)^	100
	4	20.534(1.54) ^(e,f)^	100
Z group (Z-PRIME plus)	1	10.542(1.51) ^(g,h,C)^	100
	2	13.346(1.53) ^(A)^	100
	3	11.574(1.34) ^(g,h,B)^	100
	4	11.320(1.53) ^(g)^	100

Identical, same capital letters indicate no statistically significant differences ( *p* > 0.05) between groups. Identical, same lowercase letters indicate no statistically significant differences ( *p* > 0.05) in the same group.

In the C, T, MN, and Z groups, applying the primer twice had the strongest shear bond strength compared with the other application numbers in each group, with a significant difference found ( *p* < 0.05) in the T, MN, and Z groups. However, in the MC group, the shear bond strength was significantly lower after two applications than it was after one application ( *p* < 0.05). There was no significant difference in shear bond strength ( *p* > 0.05) between the 3 and 4 applications in all the groups, and both had significantly lower shear bond strengths than the 2 applications in each group ( *p* < 0.05).

The single application T group had the strongest shear bond strength, followed by the C, MN, MC, Z, and control groups, with no significant difference ( *p* > 0.05) between the MN, MC, and Z groups.

**Fig. 2:**
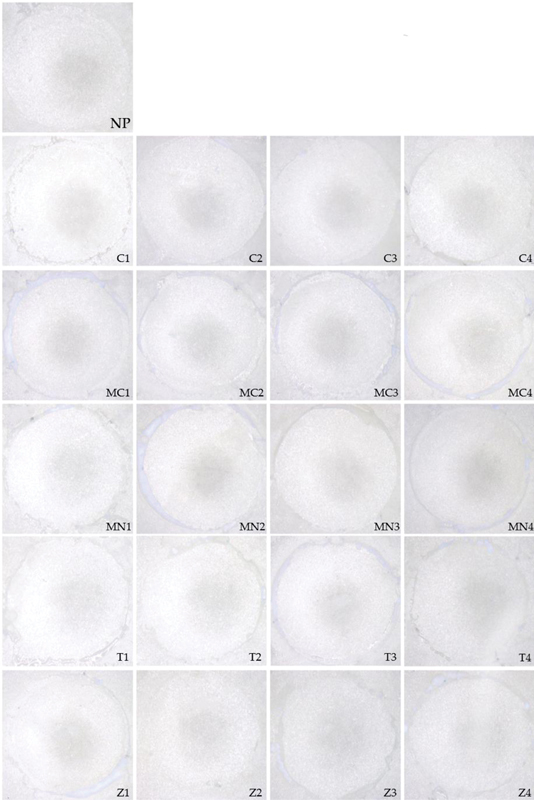
Adhesive failure mode illustrating (NP) No primer application (control); (C) Clearfil ceramic primer plus; (MC) M&C PRIMER; (MN) Monobond N; (T) Panavia V5 Tooth Primer; (Z) Z-Prime Plus. Number: (1) Applied one time; (2) Applied two times; (3) Applied three times; (4) Applied four times.

The failure mode analysis results are seen in Table 2 . In every group, every specimen demonstrated adhesive failure ( Fig. 2 ). There were no cases of cohesive or mixed failure.

The mean concentrations of phosphorous determined using ICP-OES in each brand of phosphate-containing primer are illustrated in Table 3 . The results demonstrated that the T group had the highest concentration followed by the C, MN, Z, and MC groups, with no significant difference ( *p* > 0.05) between the MN and Z groups.

**Table 3:** ICP-OES results of phosphorous concentration in the MDP-containing primers

Groups	Concentration (SD) (mg/L)
C group (CLEARFIL CERAMIC PRIMER)	5.0142 (0.27) ^a^
MC group (M&C PRIMER)	0.8425 (0.02) ^b^
MN group (Monobond N)	2.7337 (0.08) ^c^
T group (PANAVIA V5 Tooth Primer)	32.5100 (1.88) ^d^
Z group (Z-PRIME plus)	2.0739 (0.19) ^c^

Identical, same letters indicate no statistically significant differences ( *p* > 0.05) between groups.

## Discussion

The objectives of this study were to establish the failure characteristics and to ascertain the impact of the number of applications of each MDP-containing primer brand on the shear bond strength of zirconia-based dental ceramic to resin cement. The results indicated that the bond strength of zirconia was affected by the number of applications of each MDP-containing primer brand. In all primers except the M&C PRIMER, two applications generated the highest shear bond strength and declined when three and four applications were used, and more than three applications did not significantly change the shear bond strength. Thus, the null hypothesis that the effect of each number of applications for each MDP-containing primer brand on bond strength is not different was rejected.

Due to the structural stability of zirconia, the bonding effectiveness of various materials to zirconia is impaired, therefore treating the zirconia surface prior to cementation is crucial when bonding to zirconia. 17 A variety of mechanical and chemical surface treatment techniques have been used to improve the adhesion of zirconia. 4 5 Studies have demonstrated that sandblasting is necessary for zirconia bonding during mechanical surface treatments. 18 19 20 However, phosphate functional monomer has also been shown to improve the bond strength when used as a chemical surface treatment for zirconia-based restorations. 21 22 and several studies have demonstrated that the combination of sandblasting and phosphate functional monomer results in a greater shear bond strength than either technique alone. 10 11 23 Zirconia that has had its surface roughened is treated with conditioners that contain MDP to chemically bond zirconia to methacrylate-based composites. The MDP molecule contains a functional ethylene end group, which functions as a crosslinking agent with unsaturated C = C bonds in the composite cement, and a functional phosphoric acid end group, which promotes adhesion. Through hydrogen bonding or ionic connection between P-OH and Zr-OH or between P-O and partially positive Zr, these molecules will continue to be in contact with zirconia. 24

Our results revealed that not applying a primer generated the lowest shear bond strength of all the groups. These results suggest that using an MDP-containing primer before cementation achieves better results compared with using cement alone. The reasons for this include the chemical bonding of MDP, a phosphate monomer, to the zirconia's surface. 9 25 26 Stronger bonds are created between the zirconia and the resin cement due to the phosphate group on one end of MDP chemically reacting with the oxide layer, and the double-bonded methacrylate on the other end polymerizing with the cement. 24 26

The study's results indicate that two, three, and four applications of phosphate-based primer generated a higher shear bond strength compared with one application in the T, Z, and MN groups because the concentration of the functional monomer may have increased as a result of multiple primer applications. 13 Thus, higher concentrations of MDP make stronger bonds. 9 24 27 28

The bonding between zirconia and resin cement is strengthened due to the increased formation of zirconia oxide and phosphate bonds. 8 26 29 Another factor could be the greater solvent evaporation that occurred as additional primer applications were used. The CPP used in this study as a solvent contains ethanol 80% by weight. 29

We also found that two applications established the highest shear bond strength in all primers, except the MC primer, because the bond strength depends on the intensity of the connection between the substrate and adsorbate. The adsorption of molecules to a substrate is frequently referred to as physisorption or chemisorption. When a primer is applied, physisorption and chemisorption both occur. Weak electrostatic interactions, such as Van Der Waals interactions, dipole-dipole forces, and London forces, will lead to physisorption. These interactions are the weakest and most easily broken. Chemisorption occurs when the adsorbate bonds covalently to the substrate through electron sharing or transfer. Chemisorption interactions are typically two orders of magnitude stronger than physisorption interactions. Therefore, more electron transfer or sharing might occur after the second application of primer than after the first application.

We also observed that three and four applications of a primer containing phosphate resulted in a shear bond strength that was significantly lower compared with two applications. This might be because the phosphate functional monomers had bonded with the entire zirconia oxide layer 13 or it could imply that chemisorption interactions occur throughout the entire zirconia oxide layer, and when more primer is applied, physisorption will occur more than chemisorption and cover the chemisorption-bonded layer, making the bonded layer the weakest point.

The study's results demonstrated that T has the strongest shear bond strength of all the groups. This is because T contains the significantly highest phosphorous concentration compared with the other primers. As mentioned above, higher MDP concentrations cause more bonds to form between phosphate and zirconia oxide, strengthening the bonds between zirconia and resin cement. 24 Therefore, the bond strengths exhibited by each group can be explained by their respective phosphorous concentrations. Furthermore, the bond strength may depend on the quality, or more specifically the purity of MDP according to Yoshihara et al. (2015). 30

This study's findings in which MC generated the highest shear bond strength after its first application and decreased after the second, third, and fourth applications might be because it has acetone as a solvent. This is because acetone has a higher rate of solvent evaporation than other solvents. According to Garcia et al, the vapor pressure of acetone, methanol, ethanol, and water is 185, 120, 54, and 23 mm Hg, respectively 31 thus, acetone can undergo a greater amount of chemical reaction on the zirconia surface. It is also possible that the phosphate–functional monomers chemically reacted with the entire zirconia oxide layer present in the first application. 13 Alternately, the acetone evaporation might cause chemisorption interactions to occur everywhere on the zirconia surface. However, applying additional primer causes physisorption to predominate and cover the chemisorption-bonded layer, rendering the bonded layer the weakest part of the material.

Due to the limitations of this study, the authors did not investigate the effect of thermal cycling (simulated aging) on the bonding strength. As a result, after the suggested changes, further study is required concerning thermal cycling. In addition, because the oral cavity contains a variety of environments, such as saliva and pH level changes, which could significantly affect the bond strength between the zirconia ceramic, additional research is required to evaluate these parameters.

## Conclusions

Within the limitations of this study, the number of applications of each MDP-containing primer brand affected zirconia bond strength. Two applications produced the strongest shear bond strength in all primers except the M&C PRIMER, while three and four applications decreased it. However, the combination of sandblasting and MDP-containing primer is crucial for achieving strong bonding with zirconia.
